# Supplementary material for: The Global Demand and Supply Balance of the Human Papillomavirus Vaccine: Implications for the Global Strategy for the Elimination of Cervical Cancer
Source: Vaccines (Basel). 2023 Dec 19;12(1):4. doi: 10.3390/vaccines12010004 (PMC10820532; doi:10.3390/vaccines12010004)
Supplement: Supplementary file 1 [file vaccines-12-00004-s001.zip › vaccines-2699869-supplementary.pdf]

Demand Forecasts

For Gavi-eligible countries, Gavi demand estimates were used directly in the demand model.[1] The Gavi estimates included routine and MAC demand for 72 LICs and MICs. These did not include India. For the remaining countries, vaccine demand was estimated as shown in Figure A1. For India, demand was estimated on a state-by-state basis and then consolidated nationally. Routine immunization was estimated for all non-Gavi countries on a country-by-country basis. MAC demand was included in the Gavi forecast with the exceptions of France and Japan, which are expected to conduct MACs between 2022 and 2024; the figure for those countries were estimated separately, consistently with the methodology presented in Figure A1. Private market vaccine demand was estimated for MICs where the HPV vaccine is not included in the national immunization schedule. The estimate was based on the portion of the population in the wealthiest income bracket. For the MICs where the HPV vaccine is included in the national schedule, private sector demand was not distinguished from public market demand and was captured in the overall forecast. Private market demand forecasts for China were based on local retrospective vaccine lot release information.[2]

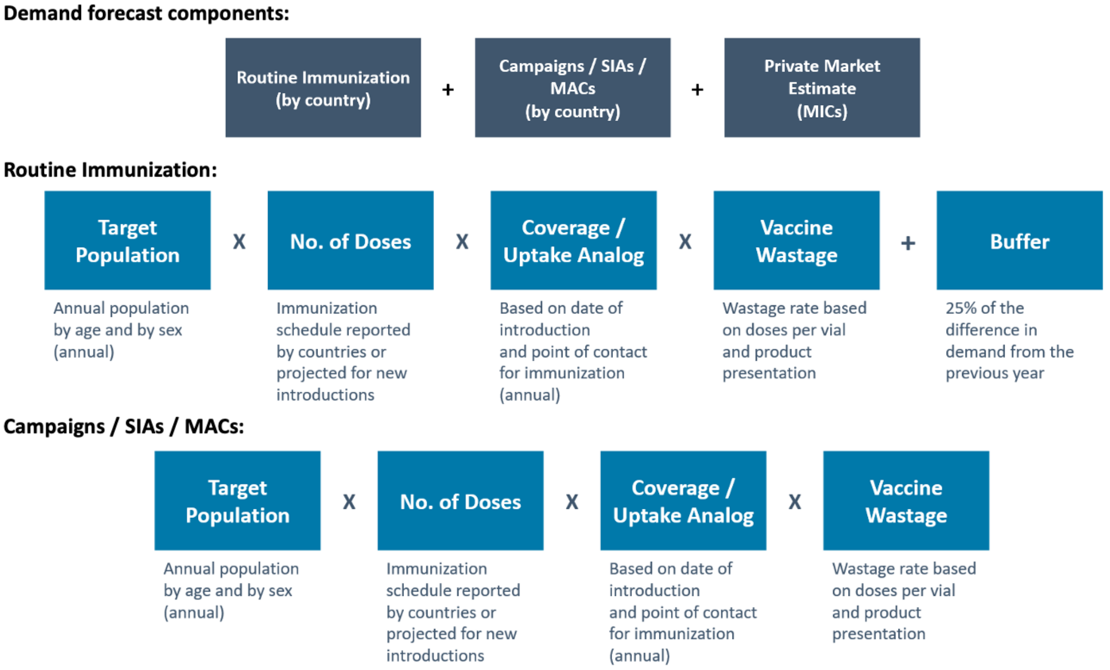

Figure S1. Demand calculations.

Demand components were calculated as follows:

The **target population** for future demand was 9–14-year-old girls (9-year-old cohort for routine, over all five years for MACs). Boys at 9 years of age were included for countries with an existing gender-neutral immunization policy.[3] Opportunistic catch-up (immunization of older adolescents and adults) was incorporated into the forecast via the coverage/uptake analog (see details below). United Nations Population Division WPP[4]

---

[1] Cite January 2019 version

[2] Chinese Lot Release Data can be found at: http://www.nifdc.org.cn/CL0108/

[3] Countries currently recommending immunization of boys as well as girls: Antigua and Barbuda, Argentina, Australia, Austria, Bahamas, Barbados, Belgium, Belize, Bhutan, Brazil, Canada, Chile, Croatia, Czech Republic, Denmark, Finland, France, Germany, Grenada, Guyana, Hungary, Ireland, Israel, Italy, Luxembourg, Netherlands, New Zealand, Norway, Panama, Portugal, Qatar, Russian Federation, Saint Lucia, Slovakia, Slovenia, Sweden, Switzerland, Trinidad and Tobago, Turkmenistan, United Kingdom, United States of America, Uruguay.

[4] UN Population Division World Population Prospects (WPP): https://population.un.org/wpp/

population projections by age and by sex were used for each country. The Indian census[5] was used for the population by state.

For the **number of doses**, we assumed a base case immunization schedule of two doses, with two exceptions. China was forecasted to use three doses, sub-nationally, with a domestically produced product, from 2020 to 2024 (then a two-dose schedule nationwide in 2025). Japan was also forecasted to use a three-dose schedule through the duration of the forecast. Alternative dosing schedules were modeled in additional scenarios, as shown in Table 1. Data sources were JRF country-reported immunization schedules;[6] WHO MI4A Vaccine Purchase Data;[7] and ECDC Vaccine Schedules.[8]

The coverage/uptake analog had multiple components.

Introduction date: Planned introduction dates were as reported by countries or partners such as PAHO, Gavi, the Bill & Melinda Gates Foundation, PATH, or the Clinton Health Access Initiative. Where no plans were available, WHO regional offices and experts were consulted to advise on potential introduction dates and ultimately review the MI4A proposed introduction year. For China and India, which have domestic products in the pipeline,[9,10] projected introductions were assumed to coincide with forecasted dates of national product licensure. Data sources were the JRF, WHO Immunization Repository,[11] Gavi, HPV partners and experts, and WHO Regional Offices, with final review by the MI4A Advisory Group.

Coverage: Country-specific estimates are sourced from WHO HPV coverage data (as of July 2021) and were the midpoint of the first and second dose for females. For countries with a gender-neutral schedule, male coverage data were used where available or 90% of female coverage was applied, which was based on WHO analysis of HPV coverage differentials between females and males in countries with gender-neutral schedules.

Coverage in non-Gavi countries for future introductions and for countries for which coverage data were not available: Regional coverage estimates were calculated by averaging coverage values by WHO region for all countries with more than one year of national introduction, based on available data from 2020 (Table A1). These estimates were then applied for future introductions. Coverage estimates for the Americas incorporated a PAHO RF input into annual procurement.

Table S1. Regional coverage estimates applied to future introductions.

Region	Coverage/Uptake Analog
AFR	70%
AMR	70%
EMR	65%
EUR	75%
SEAR	80%
WPR	80%

[5] Indian census (2011), population by state, by age, and by sex: http://censusindia.gov.in/

[6] JRF country-reported immunization schedules: http://apps.who.int/immunization_monitoring/globalsummary/schedules

[7] MI4A vaccine purchase data: https://www.who.int/teams/immunization-vaccines-and-biologicals/vaccine-access/mi4a/mi4a-vaccine-purchase-data

[8] ECDC vaccine schedules: https://vaccine-schedule.ecdc.europa.eu/

[9] Subnational, non-reimbursed EPI use of HPV in China was forecasted between 2022 and 2024. National introduction was projected for 2025.

[10] Introduction year forecasted by state for India, phased between 2024 and 2026.

[11] WHO Immunization Repository: https://www.who-immunization-repository.org/

Uptake: To reflect the gradual increase in coverage in the first few years after new HPV vaccine introduction, coverage of the primary target population for new introductions was scaled down as follows: Year 1: 100%, Y2: 80%, Y3: 90%, Y4+: 100%. For Gavi-supported countries, the outputs directly from the SDS were used; therefore, uptake assumptions for these countries were not generated.

Coverage increases over time. For the base case and Scenarios A–C, coverage was forecasted to increase 0.5% per year in all countries until they reached 90% coverage, starting in the fifth year following HPV introduction into the routine immunization programme. For Scenario D, coverage was forecasted to increase linearly from the current coverage or from the first year of introduction as needed for all countries to achieve 90% coverage by 2030.

Coverage adjustment for catch-up of older populations: Opportunistic catch-up of older populations is particularly relevant for HICs with lower coverage in the target age cohort. This additional demand is accounted for by adding the estimated coverage in older age groups to the estimated coverage in the target cohort. For example, if the forecasted coverage of the target cohort is 65%, and forecasted additional coverage through opportunistic catch-up is 10%, the coverage analog used to forecast annual demand will be 75%. Additional coverage estimates were based on evidence available in the literature, when available. When not available, estimates were made based on the World Bank income group classification of each country. The opportunistic catch-up coverage assumptions were the following: 10% for USA, 6% for all other HICs, 3% for UMICs, and 0% for all other MICs/LICs.

Coverage adjustment for COVID-19 impact: The impact of COVID-19 on HPV coverage was also factored into the coverage analogs for each country. WHO IVB data on HPV vaccine coverage from 2019 were used as the pre-pandemic reference year for each country's HPV vaccine coverage. If no drop in HPV coverage was observed from 2019 to 2020, coverage was assumed to be stable at 2019 levels through 2023, before coverage growth resumed in 2024. If a drop in HPV coverage was observed from 2019 to 2020 and 2021 coverage data were not available, coverage in 2022 was calculated as an absolute reduction of 10% from 2019 HPV coverage before full recovery in 2023 and coverage growth starting in 2024.

Wastage: For routine immunization, wastage was assumed to be 5% for one-dose vials and 10% for two-dose vials. Wastage for MACs was assumed to be 5%.

Buffer: This was set as 25% of the difference in demand from the previous year.
